# Ventricular fibrillation arrest after blunt chest trauma in a 33-year-old man, commotio cordis?

**DOI:** 10.1186/s12872-022-02689-4

**Published:** 2022-06-03

**Authors:** Neha Patel, Clarissa Pena, Zeid Nesheiwat, Fnu Zafrullah, Ehab Eltahawy

**Affiliations:** 1 Department of Internal Medicine, The University of Toledo, 3000 Arlington Avenue, Toledo, OH 43614 USA; 2 Department of Cardiovascular Medicine, The University of Toledo, 3000 Arlington Avenue, Toledo, OH 43614 USA; 3 Department of Internal Medicine, The University of Toledo, 2100 W. Central Ave, Toledo, OH 43614 USA

**Keywords:** Commotio cordis, Ventricular fibrillation, Arrythmia, Non-penetrating trauma, Athlete, Sudden cardiac death

## Abstract

**Background:**

Commotio cordis is an event in which a blunt, non-penetrating blow to the chest occurs, triggering a life-threatening arrhythmia and often sudden death. This phenomenon is often seen in young, male athletes and has become increasingly well-known over the past few decades. We present a unique case in which ventricular fibrillation occurs in an older male athlete after blunt trauma.

**Case presentation:**

Patient with no known medical history was brought to the ER after being found unconscious after a soccer ball kick to the chest. He was found to be in ventricular fibrillation and successfully resuscitated on the soccer field. Patient was admitted to the hospital and lab workup and initial imaging were unremarkable, except elevated troponin and lactate, which returned to normal levels. An echocardiogram showed global left ventricular systolic dysfunction with a visually estimated ejection fraction of 45–50%. Coronary showed angiographically nonobstructive coronary arteries. The patient was diagnosed with commotio cordis and discharged from the hospital in stable condition. Follow-up echocardiogram continued to show low ejection fraction and event monitor demonstrated frequent polymorphic ventricular tachycardia with periods of asystole.

**Conclusion:**

This case is unique in that blunt trauma to the chest from a soccer ball immediately triggered ventricular fibrillation in a patient with a possible cardiomyopathy. It is possible that the blunt trauma caused primary commotio cordis that led to cardiomyopathy in a previous healthy man, or that an underlying cardiomyopathy made it more likely for this to occur. Overall, increased awareness and prevention efforts of blunt chest trauma are required to reduce the high mortality associated life-threatening arrhythmias. There is limited data regarding the interplay between these two entities.

## Introduction

Commotio cordis is defined as sudden cardiac arrest secondary to blunt, nonpenetrating trauma to the chest wall. An important distinction between commotio cordis and cardiac contusion is that commotio cordis is due to blunt chest wall trauma that does not cause structural cardiac damage but instead leads to an electrical event [1, 2]. The most frequent arrhythmia occurring with commotio cordis is ventricular fibrillation (VF), however polymorphic ventricular tachycardia and heart block have also been reported [1]. Higher energy impacts directly over the precordium are more likely to cause VF with ST segment elevation mediated by an increase in potassium current across the cell membrane via potassium ATP channel [3–7]. These impacts occur 10–30 ms prior to the T wave peak, resulting in VF [4, 8]. Most individuals affected by commotio cordis will not survive the cardiac arrest with resuscitation attempts being successful in only 35% of patients affected [2, 3]. The majority of patients affected by commotio cordis are young male athletes with decreasing cases occurring over the age of 20. The mean age of individuals affected by this condition is 14 years old, with 78% < 18 years of age. The most frequently affected athletes are those participating in baseball, softball, hockey, and lacrosse [1, 4]. We present a case of a 33-year-old male who suffered a blow to the chest while playing soccer that led to VF arrest and was subsequently found to have cardiomyopathy. The diagnosis of commotio cordis is possible.

## Case presentation

**Fig. 1:**
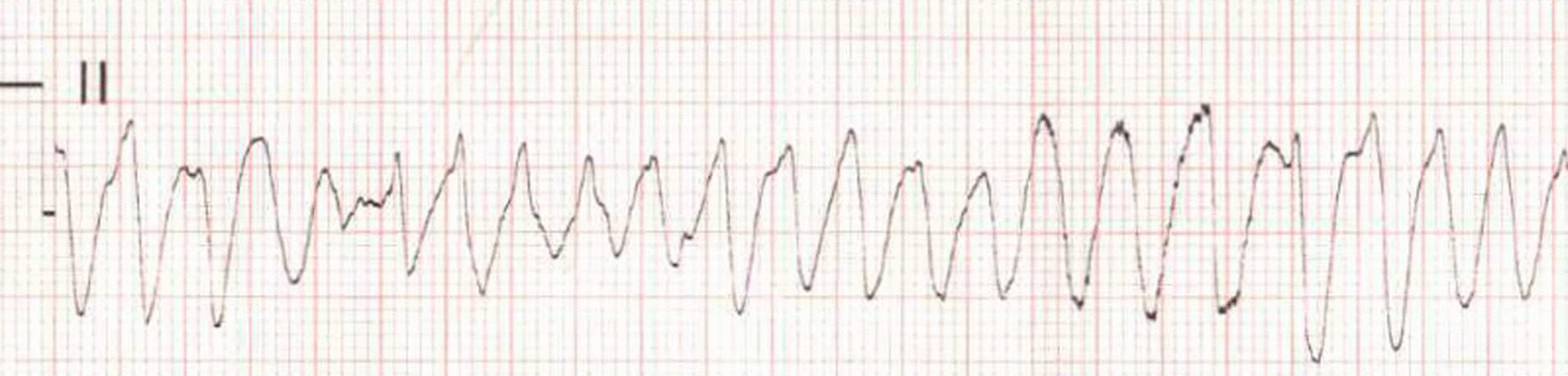
Initial rhythm strip showing ventricular fibrillation

A 33-year-old African American male with no past medical history presented to the emergency room after suffering cardiac arrest. The patient was playing soccer and suffered blunt trauma to the chest from a forceful soccer ball kicked directly at his chest. He walked a few steps after the blunt force, then about 30 s later, he collapsed and was described as having several jerking movements. Fortunately, medically trained personnel were involved in the soccer match, witnessed the event, and immediately performed CPR. He was found to be apneic and in VF arrest. Three rounds of cardiopulmonary resuscitation (CPR) were completed with a total of three defibrillation attempts and one dose of epinephrine administered. A rhythm strip in the ambulance showed VF (Fig. 1). Return of spontaneous circulation was achieved, the patient regained consciousness, and his rhythm reverted to normal sinus rhythm.

On arrival to the ER, he was mildly confused but following commands and answering questions appropriately. His only complaint was mild chest discomfort, exacerbated by palpation and deep breaths. His last recollection was playing soccer prior to waking up in the ambulance. He denied ever experiencing exertional syncope, exertional chest pain, or a prior history of sudden cardiac arrest. He was otherwise healthy and was not taking any long-term medications. He denied any family history of sudden cardiac death, cardiac arrhythmia, or unexplained syncope. He denied any alcohol, tobacco, or drug use. Physical exam was unremarkable except for tenderness to palpation in pre-sternal region.

**Fig. 2:**
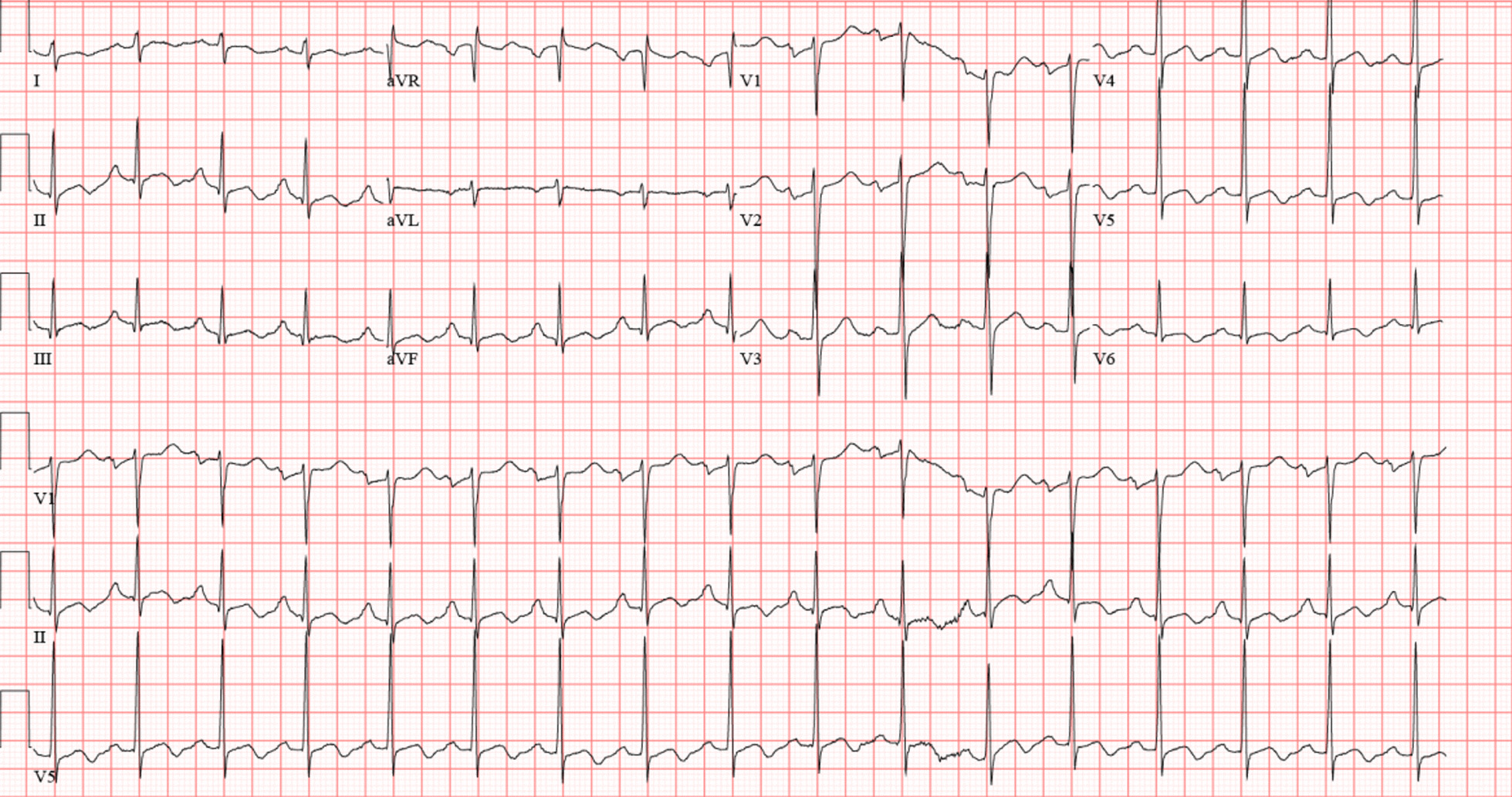
EKG on arrival to emergency room showing sinus rhythm with QT prolongation (490 ms)

Initial labs showed potassium of 2.9 mEq/L (3.5–5.1), creatinine 1.56 mg/dL (0.7–1.3), magnesium 2.0 mg/dL (1.9–2.7), troponin 0.04 ng/mL (0.0–0.04), lactate 9.2 mmol/L (0.5–2.2), creatinine kinase 574 IU/L (30–223), SARS COV2 antigen negative, tox screen negative. His initial EKG showed sinus rhythm, right atrial enlargement, right axis deviation, QT prolongation (QTc 490 ms), and T wave abnormalities (Fig. 2). Computed tomography (CT) brain and CT abdomen/pelvis were unremarkable. CT angiography (CTA) chest showed alveolar opacifications suggesting pulmonary edema but no evidence of pulmonary embolism. Potassium was replaced intravenously, and he was given 1 L fluid bolus initially in the ER. Cardiology was consulted due to cardiac arrest with unclear etiology.

**Fig. 3:**
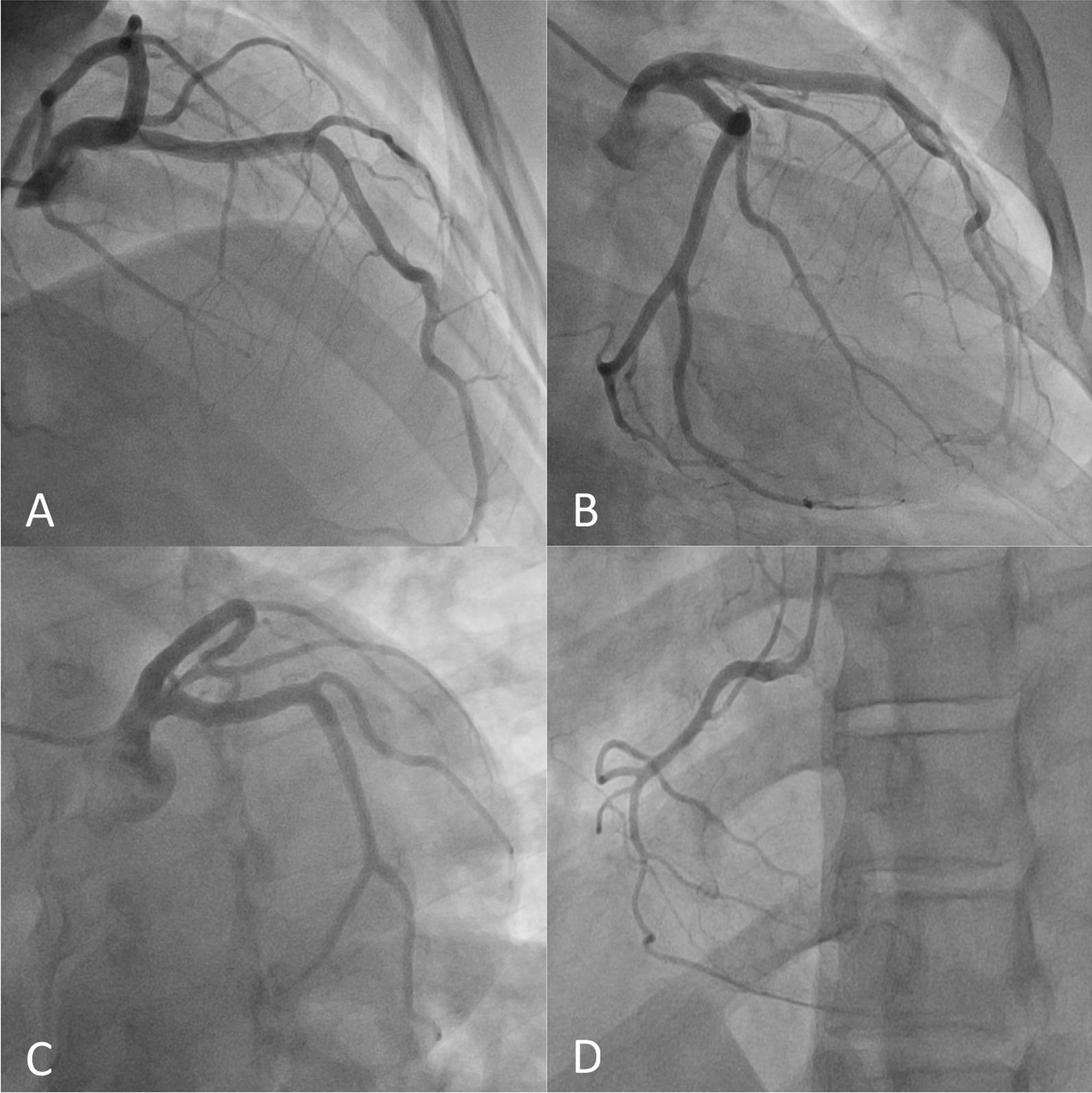
**A** RAO cranial view, **B** RAO caudal view, **C** LAO caudal view, **D** RCA view. All views demonstrating non-obstructive coronaries

An echocardiogram showed global left ventricular systolic dysfunction with a visually estimated ejection fraction of 45–50%. There was basal inferolateral, basal anterior lateral, mid inferolateral and apical lateral hypokinesis. No left ventricular hypertrophy was noted. Troponin increased to 1.43 ng/mL and electrocardiogram (EKG) showed sinus rhythm with improvement in inferior T wave abnormalities. Due to these findings, coronary angiography was performed; this showed angiographically nonobstructive coronary arteries (Fig. 3).

**Fig. 4:**
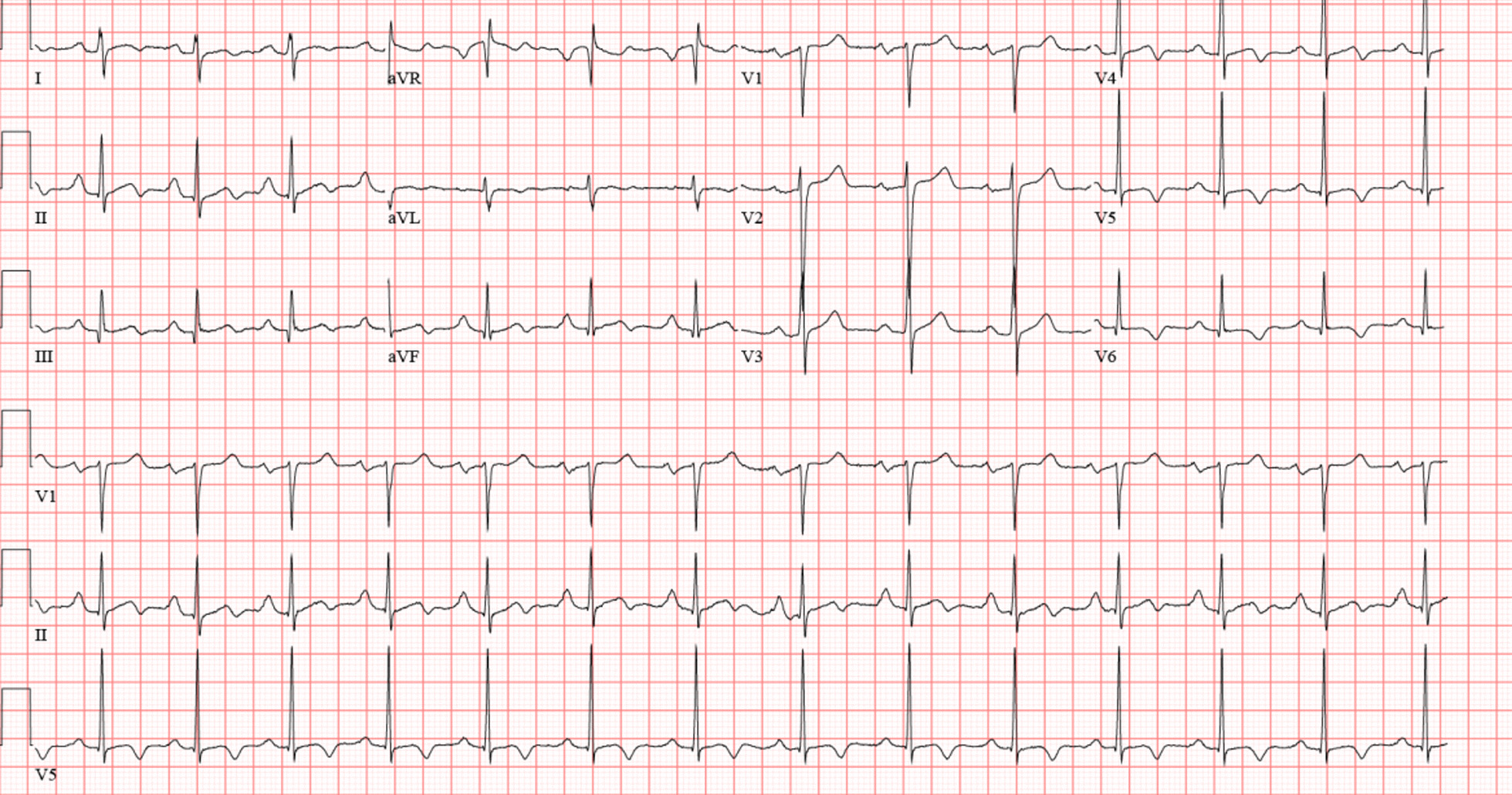
Follow up EKG showing sinus rhythm with improved QT prolongation (448 ms)

The following day, lactate, electrolytes, and creatinine normalized, and a downtrend in troponin was noted. Repeat EKG showed improvement in QTc to 448 ms (Fig. 4). Based on these findings, the patient was diagnosed with commotio cordis as the likely cause of his VF arrest. He was discharged in a stable condition.

Patient followed up 1 month later and was found to have normal potassium level (4.3 mEq/L), normal chest x-ray, and echocardiogram showing persistent reduced ejection fraction (35–40%). He was started on beta blocker and angiotensin-converting enzyme inhibitor (ACE-inhibitor); however, spironolactone was not initiated due to concern for low blood pressure. In addition, event monitor revealed frequent episodes of polymorphic VT and periods of asystole. The patient was referred for implantable cardioverted defibrillator (ICD) implant. Cardiac magnetic resonance imaging (MRI) has been ordered but not yet performed.

## Discussion

In this case, an African American adult male suffered a blow to the chest by a soccer ball and found to be in ventricular fibrillation. It is suspected that the patient experienced commotio cordis. Several factors important in facilitating commotio cordis include the location of the blow, the velocity, and the timing within the cardiac cycle [1]. In this patient, the soccer ball impact was directly to the chest, and he was subsequently found to be in ventricular fibrillation shortly after the impact.

There are several unique factors in our case; one of which is the patient’s age. Generally, commotio cordis is a condition that affects young male athletes. Children/teenagers tend to have more pliable chest walls that facilitate transmission of energy from the chest impact to the myocardium. While less frequent, commotio cordis has occurred in adults, who have more developed and less malleable chest walls [9]. The decreased incidence of commotio cordis in adults may also be due to the higher velocities required to generate the same sudden rise in intracavitary pressure. Optimal projectile velocity for ventricular fibrillation induction through commotio cordis is 40 miles per hour (mph) in a small swine model (8–25 mg) [10]. One case reports commotio cordis in which an adult was struck with a very high velocity projectile, at nearly 200 mph. In our case, the soccer ball would have needed to hit the chest with a high enough velocity to overcome the less malleable chest and cause ventricular fibrillation. The rarity of commotio cordis in adults is also likely due, in part, to the significantly higher velocities that may be required to generate the same sudden rise in intracavitary pressure.

While this patient did not report any cardiac history, no records could be found to indicate if the patient had any previous cardiac workup, such as an EKG, prior to presentation. While commotio cordis has been found in patients with no underlying cardiac dysfunction, there has been a case of a previously healthy 35-year-old male diagnosed with commotio cordis who presented 9 months later with wide complex tachycardia. It was hypothesized that a myocardial scar caused the episode of commotio cordis [11]. Similarly, the patient we presented may have had an underlying condition that allowed this event to occur in an otherwise healthy person. Given the T wave inversions in the lateral precordial leads in the initial and follow up EKG, we suspect this patient had an underlying cardiomyopathy that may have predisposed him to an arrhythmia following the blunt trauma. There has also been an association of a genetic predisposition with normal QT interval at baseline but with a susceptibility to prolonged QT with an insult such as blunt force trauma affecting repolarization [1]. While underlying arrhythmias do not automatically preclude participation in sports, if the ventricular tachyarrhythmias are frequent, that may necessitate withdrawal from sports as there is an association with sudden cardiac death. Without previous electrocardiograms or Holter monitor readings, instances of premature ventricular depolarization occurring prior to the blunt chest trauma that could have predisposed to VF arrest cannot be excluded [12, 13]. The cardiac MRI will aid in elucidating an explanation of the patient’s cardiomyopathy.

Data show that nearly 60% of individuals survive commotio cordis in recent years, and each year the survival rates are improving. However, it has been found that African Americans are less likely to survive than their Caucasian counterparts (4% vs 33%), partially attributed to delays in resuscitation (44% vs 22%) and less frequent use of automated external defibrillators (4% vs 8%) [13]. Higher survival rates have been noted in affected individuals receiving prompt resuscitation with onsite automated defibrillator use [13]. The standardization of resuscitation out of the hospital has seen a steady increase in survivors from all types of cardiac arrest, including commotio cordis. The main reason for improved outcomes in these patients, including the patient in this case, is due to a combination of increased awareness and access to medical care and public defibrillators, as well as timely resuscitation.

## Conclusion

In this case, commotio cordis is a possible explanation for the patient’s presentation. Commotio cordis is an uncommon, yet potentially fatal cardiac condition. While this case is unusual in that commotio cordis is generally seen in young athletes, this case demonstrates that it should be considered in this population under the appropriate circumstances, particularly in individuals with pre-existing cardiomyopathy. While survival rates have been improving, the mortality rate remains high. The implication of high mortality rate is that not enough case studies and data are available to determine if commotio cordis can cause cardiomyopathy. Further longitudinal studies should be completed following the survivors of commotio cordis to determine if long term cardiomyopathies can arise. Given its frequent occurrence during sports, recommendations including use of chest wall protectors have been implemented; however, unfortunately this effort has failed to demonstrate a decrease in incidence [14]. Continued efforts by the medical community and sporting organizations to increase awareness of this condition in both children and adults is required to identify commotio cordis and improve outcomes.

## Data Availability

Data sharing not applicable to this article as no datasets were generated or analyzed during the current study.

## Abbreviations

VF: Ventricular fibrillation
ATP: Adenosine triphosphate
CPR: Cardiopulmonary resuscitation
CT: Computed tomography
CTA: CT angiography
ACE-inhibitor: Angiotensin-converting enzyme inhibitor
ICD: Implantable cardioverted defibrillator
MRI: Cardiac magnetic resonance imaging
mph: Miles per hour
